# Crystal Structures of Bat and Human Coronavirus ORF8 Protein Ig-Like Domain Provide Insights Into the Diversity of Immune Responses

**DOI:** 10.3389/fimmu.2021.807134

**Published:** 2021-12-17

**Authors:** Xiaoxue Chen, Zhechong Zhou, Chunliu Huang, Ziliang Zhou, Sisi Kang, Zhaoxia Huang, Guanmin Jiang, Zhongsi Hong, Qiuyue Chen, Mei Yang, Suhua He, Siqi Liu, Jie Chen, Kenan Li, Xin Li, Jing Liao, Jun Chen, Shoudeng Chen

**Affiliations:** ^1^ Molecular Imaging Center, Guangdong Provincial Key Laboratory of Biomedical Imaging, The Fifth Affiliated Hospital, Sun Yat-sen University, Zhuhai, China; ^2^ Zhongshan School of Medicine, Sun Yat-sen University, Guangzhou, China; ^3^ Department of Oral Emergency and General Dentistry, Affiliated Stomatology Hospital of Guangzhou Medical University, Guangdong Engineering Research Center of Oral Restoration and Reconstruction, Guangzhou Key Laboratory of Basic and Applied Research of Oral Regenerative Medicine, Guangzhou, China; ^4^ Department of Clinical Laboratory, The Fifth Affiliated Hospital of Sun Yat-sen University, Zhuhai, China; ^5^ Department of Infectious Disease, The Fifth Affiliated Hospital, Sun Yat-sen University, Zhuhai, China; ^6^ Department of Gastroenterology, The Fifth Affiliated Hospital of Sun Yat-sen University, Zhuhai, China; ^7^ Guangdong Institute of Gastroenterology, The Sixth Affiliated Hospital, Sun Yat-sen University, Guangzhou, China

**Keywords:** SARS-CoV-2, COVID-19, accessory protein, ORF8, RaTG13, monocytes, crystallography

## Abstract

ORF8 is a viral immunoglobulin-like (Ig-like) domain protein encoded by the severe acute respiratory syndrome coronavirus 2 (SARS-CoV-2) RNA genome. It tends to evolve rapidly and interfere with immune responses. However, the structural characteristics of various coronavirus ORF8 proteins and their subsequent effects on biological functions remain unclear. Herein, we determined the crystal structures of SARS-CoV-2 ORF8 (S84) (one of the epidemic isoforms) and the bat coronavirus RaTG13 ORF8 variant at 1.62 Å and 1.76 Å resolution, respectively. Comparison of these ORF8 proteins demonstrates that the 62-77 residues in Ig-like domain of coronavirus ORF8 adopt different conformations. Combined with mutagenesis assays, the residue Cys20 of ORF8 is responsible for forming the covalent disulfide-linked dimer in crystal packing and *in vitro* biochemical conditions. Furthermore, immune cell-binding assays indicate that various ORF8 (SARS-CoV-2 ORF8 (L84), ORF8 (S84), and RaTG13 ORF8) proteins have different interaction capabilities with human CD14^+^ monocytes in human peripheral blood. These results provide new insights into the specific characteristics of various coronavirus ORF8 and suggest that ORF8 variants may influence disease-related immune responses.

## Introduction

The coronavirus disease 2019 (COVID-19) pandemic caused by severe acute respiratory syndrome coronavirus 2 (SARS-CoV-2) has posed substantial global challenges to health and economic systems (https://covid19.who.int/). The rapid and persistent mutations of SARS-CoV-2 triggered a new phase of the pandemic outbreak (1–4). Although the accessory proteins of SARS-CoV-2 appear not necessary for virus replication, several mutations have potentially contributed to increasing the pathogenesis and transmissibility of the virus (5) (https://covariants.org/).

Immunoglobulin-like (Ig-like) domain proteins play crucial roles in mediating macromolecular interactions in the immune system (6–8). Viruses evolve Ig-like domain proteins to evade the host immune system, inhibiting the host immune response (9, 10). SARS-CoV-2 ORF8 is confirmed as an Ig-like fold containing accessory protein, which may be involved in a potential evolutionary arms race between virus and host (11, 12). The clinical effect of a 382-nucleotide deletion (Δ382) in SARS-CoV-2, an ORF8 deleted variant, appears to be a milder infection with the less systemic release of pro-inflammatory cytokines (13). ORF8 has been detected as a secreted protein and is highly immunogenic in COVID-19 patients (14, 15). A deep understanding of ORF8 may provide new clues into the pathogenesis of SARS-CoV-2. The predominant ORF8 natural variants include Leu84 and Ser84 (16). The S84 variant in the ORF8 protein is considered related to mild disease among the clinical outcomes (17).

The ORF8 protein is identified as a fast-evolving protein. It has only been found in lineage B betacoronaviruses, including SARS-CoV-2, SARS-CoV (severe acute respiratory syndrome coronavirus), and bat coronavirus RaTG13 (a closely related bat betacoronavirus of SARS-CoV-2) (11, 12, 16–18). ORF8 genes in SARS-CoV-2-related coronaviruses (SARS2r-CoVs) are constantly evolving in their natural reservoirs (12). RaTG13 ORF8 (UniProtKB: A0A6B9WE90) and SARS-CoV-2 ORF8 L84 (UniProtKB: P0DTC8) share 95% amino acid sequence identity. Whether and how these differences are involved in SARS2r-CoVs interacting with the host are not fully clarified.

Previous scRNA-seq data of peripheral blood mononuclear cell (PBMC) samples in COVID-19 patients indicated that monocytes and megakaryocytes are considered critical peripheral sources of cytokine storms (19). Monocytes are antigen-presenting cells that load antigens on MHC class I and II molecules and prime CD8^+^ and CD4^+^ T cells. Furthermore, monocytes have both pro-inflammatory and anti-inflammatory properties (20). The latest study demonstrates that ORF8 impairs the antigen presentation system by mediating the downregulation of MHC-I (21). However, the direct interaction of ORF8 with leukocytes remains unclear.

Here, we report the crystal structures of the ORF8 Ig-like domain from SARS-CoV-2 (ORF8 with the S84 variant) and RaTG13. These two ORF8 proteins share similar Ig-like fold conformations, characterized by forming a homodimer through a Cys20-Cys20 disulfide bond and producing an inserted area of 46-83 residues. The comparisons of the two SARS-CoV-2 variants (L84 and S84) and RaTG13 ORF8 structures, as well as the abilities of their interaction with human CD14^+^ monocytes, indicated that the ORF8 variants might affect the immune system by modulating the recognition of viral antigens with monocytes.

## Materials and Methods

### Expression Plasmids

The SARS-CoV-2 ORF8 (L84) (UniProt ID: P0DTC8) gene-encoding plasmid was a gift from Prof. Peihui Wang from Shandong University and Prof. Xi Huang from Sun Yat-sen University. The SARS-CoV-2 ORF8 (L84) cDNA (Residues 16 to 121) was cloned into a modified pRSFDuet-1 expression vector with N-terminal 6x histidines-SUMO tag. The SARS-CoV-2 ORF8 (S84) variant and RaTG13 ORF8 (UniProt ID: QHR63307) expression plasmid were generated by site-directed mutagenesis. The C20A, C20K, and the 71 IQYIDI 76 to 71 AAAAAA 76 mutants of ORF8 variants were produced by site-directed mutagenesis of their respective wild-type plasmids.

### Protein Expression and Purification

The plasmids were transformed into *E.coli* BL21 (DE3) pLysS cells. The *E.coli* cultured in Luria-Bertani (LB) media containing kanamycin at 37°C and induced with 0.2 mM Isopropyl β-D-1-thiogalactopyranoside (IPTG) at an optical density of 0.4 - 0.8. After induction, cells grew additional 4 hours at 37°C and were centrifuged to collect.

Collected cells were resuspended with lysis buffer [50 mM Tris pH 7.5, 100 mM NaCl, 2 mM β-mercaptoethanol (β-ME), 1 mM phenylmethanesulfonylfluoride (PMSF)], then lysed by a cell disruptor (JNBIO), followed by sonicating on ice. The suspension was subjected to centrifugation at 39,190 × g for at least 30 minutes at 4°C. The recombinant ORF8 protein variants were expressed in inclusion bodies. Pour off the supernatant and wash the pellet three times with wash buffer (50 mM Tris pH 7.5, 100 mM NaCl, 1 mM β-ME) containing 1%Triton X-100 and the last time without Triton X-100. The inclusion bodies were denatured in unfolding buffer A [6 M guanidine hydrochloride (Gu-HCl), 100 mM Tris-HCl, pH 7.5-8.0, 500 mM NaCl, 10 mM imidazole, 10 mM β-Me] at room temperature (RT) with rocking for 3 hours or till the pellet completely dissolved. The denatured inclusion bodies were centrifuged to remove insoluble impurities (39,190×g, 30 mins, RT). The supernatant was filtered and applied to a Ni-NTA (first Ni-NTA) column equilibrated with unfolding buffer A. After being washed with unfolding buffer A, proteins were eluted with unfolding buffer A containing 300 mM imidazole.

Two methods were applied to refold the inclusion body of ORF8 proteins. The first refolding method is salt gradient dialysis for RaTG13 ORF8 protein crystallization. The Ni-NTA purified soluble ORF8 loaded to unfolding buffer A with Ulp1 protease. Transfer the mixture sample to a dialysis bag, against 400 mL unfolding buffer B (4 M Gu-HCl, 20 mM Tris-HCl, pH 7.5, 150 mM NaCl, 10mM β-Me) at least 2 hours under vigorously stirring. Set up the dialysis apparatus at 4°C as described previously (22) (**Supplementary Figure 1**). The apparatus was a manner that the pump continuously replaces buffer from the dialysis vessel with 2 L refolding buffer A (100 mM Tris pH 7.5, 1 M L-arginine, 2 mM EDTA, 5 mM reduced glutathione, 0.5 mM oxidized glutathione, 0.2 mM PMSF). The flow rate of the peristaltic pump was calibrated to 1.5 - 2 mL/min. After the gradient has finished, optional dialyze for at least 3 h against 400 ml fresh refolding buffer A. A followed dialysis with dialysis buffer (20 mM Tris pH 7.5, 150 mM NaCl, 2 mM β-Me) to completely remove L-arginine. Because of the aggregation of protein molecules during salt gradient dialysis, we generated the second refolding method, pulse dilution, for SARS-CoV-2 ORF8 (S84) crystallization and ORF8 variant SEC analysis. A second refolding method is an optimized approach based on a previous study (11). The first Ni-NTA purified solubilized ORF8 were diluted into a 50-fold excess of cold refolding buffer B (100 mM Tris pH 8.0, 500 mM L-arginine, 2 mM EDTA, 5 mM reduced glutathione, 0.5 mM oxidized glutathione, 0.2 mM PMSF) drop-by-drop with stirring of 2 hours, followed by overnight incubation at 4°C. The refolding solution was concentrated and incubated with Ulp1 protease digestion for 6x histidines-SUMO tag removal, dialyzed overnight at 4°C against dialysis buffer.

After dialysis, Ulp1 and the uncleavaged 6xhistines-SUMO-ORF8 fusion protein were removed using a second Ni-NTA column equilibrated with dialysis buffer. Untagged ORF8 was passed through the Ni-NTA column or eluted with additional 10 mM imidazole. The ORF8 was further purified and determined by SEC using a HiLoad 16/600 Superdex 75 pg column equilibrated with a suitable buffer. The purified ORF8 was stored in 20 mM HEPES pH 7.4, 150 NaCl at -80°C.

### Crystallization, Data Collection and Structure Determination

ORF8 was crystallized by the hanging-drop vapor diffusion method at 16°C. SARS-CoV-2 ORF8 (S84) protein purified by pulse dilution refolding method contained in PBS solution with a protein concentration of 5.3 mg/ml, was mixed with an equal volume of reservoir solution [100 mM citric acid, pH 2.5, 30% (w/v) PEG6000, with a final pH 4.0]. In contrast, 5.7 mg/mL RaTG13 ORF8 protein purified by salt gradient dialysis refolding method contained in (20 mM Tris, pH 7.5, 150 mM NaCl, 1 mM DTT), was mixed with an equal volume of reservoir solution [160 mM Calcium chloride, 80 mM Sodium-HEPES pH 7.5, 23% (v/v) PEG400]. The crystals grew in drops after 3 days and were harvested at 7 to 10 days. The cryoprotectant solution contained 25% glycerol and half the concentration of the reservoir solution, respectively.

All data were collected at the Shanghai Synchrotron Radiation Facility (SSRF) BL19U1. Data integration, scaling and scale were analyzed with software HKL3000. The structures were determined by the molecular replacement method of Phenix using SARS-CoV-2 ORF8 structure (L84) (PDB ID code 7JTL) as the search mode. The model refinement was using Phenix and coot software. All structural figures were generated by PyMOL.

### Immune Cell Binding Assays

Purified recombinant SARS-CoV-2 ORF8 (L84), SARS-CoV-2 ORF8 (S84), RaTG13 ORF8, SARS-CoV-2 ORF7a, and human serum albumin (HSA) protein were coupled with a green-fluorescent dye, Alexa Fluor 488 NHS Ester (AF488, Invitrogen). Human peripheral blood mononuclear cells (PBMCs) were isolated from the peripheral blood of healthy donors. PBMCs were incubated with protein-AF488 in the 6-well plate. 2 hours later, PBMCs were collected into centrifuge tubes and washed by PBS. After that, PBMCs were incubated with human FcRs blockers (422302, BioLegend) for 20 min on ice and stained with phycoerythrin (PE) anti-human CD14 antibody (63D3, BioLegend) for 30 min on ice. Then the binding efficiencies were determined by CytoFLEX (Beckman).

## Results

### Crystal Structure of SARS-CoV-2 ORF8 (S84)

**Figure 1:**
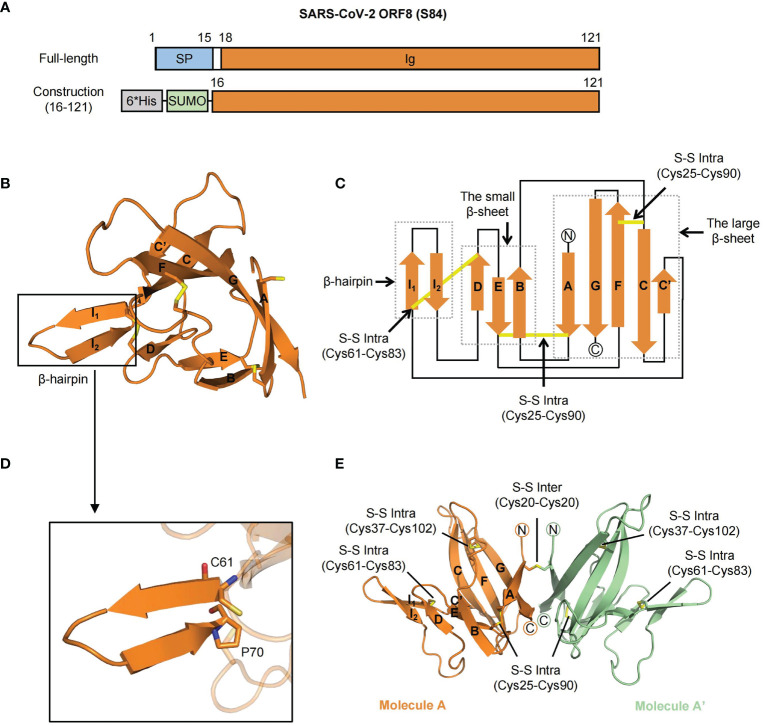
The crystal structure of SARS-CoV-2 ORF8 (S84). **(A)** Overall domain architecture of SARS-CoV-2 ORF8 (S84), including full-length and N-terminal His-SUMO tag construction for protein expression. The numbers denote residue sites of the ORF8 protein. SP, Signal peptide; Ig, immunoglobulin domain; 6*His, 6-histidine; SUMO, small ubiquitin-related modifier. **(B)** Cartoon representation of SARS-CoV-2 ORF8 (S84) Ig-like domain in one asymmetric unit. Disulfide bonds are showed with the stick model. The β-strands are assigned with the alphabet from N-terminus to C-terminus. The β-hairpin (in black box) consisting of strands βI_1_ and βI_2_ is inserted between βC’ and βD. **(C)** Topological style illustration of structure SARS-CoV-2 ORF8 (S84) Ig-like domain. The dashed boxes represent β-hairpin, the small β-sheet (βB, βD, and βE) and the large β-sheet (βA, βC, βC’, βF, βG). Disulfide bonds are showed with yellow lines. **(D)** Details of the β-hairpin of SARS-CoV-2 ORF8 (S84), which contain residues from Cys61 to Pro70. Residue Cys61 and Pro70 are shown with the stick model. **(E)** Cartoon representation of the homodimer of SARS-CoV-2 ORF8 (S84) in two symmetric units, which formed by Cys20 - Cys20 intermolecular disulfide bond. Molecule A is colored with orange, and Molecule A’ with green. Disulfide bonds are presented in yellow with the stick model, including both intermolecular and intramolecular bonds.

The full-length SARS-CoV-2 ORF8 is composed of a signal peptide (residues 1-15) and an Ig-like domain (residues 16-121) (**Figure 1A**) (12). To explore the specific characteristics of the SARS-CoV-2 ORF8 (S84) variant, we purified the Ig-like domain (ranging from 16 to 121 residues) of ORF8 (S84) and solved the crystal structure at a resolution of 1.62 Å by molecular replacement with the search model (PDB ID code 7JTL). The electron density of residues 18-121 is visible. The final refinement statistics are shown in **Table 1**.

**Table 1:** Data collection and refinement statistics.

	SARS-CoV-2 ORF8 (S84)	RaTG13 ORF8
PDB ID	7F5F	7F8L
**Data collection**	SSRF BL19U1	SSRF BL19U1
Space group	I 2 2 2	P 2_1_ 2_1_ 2_1_
Cell dimensions		
*a, b, c* (Å)	49.52, 50.19, 80.69	37.47, 49.45, 111.22
α, β, γ (°)	90.00°, 90.00°, 90.00°	90.00°, 90.00°, 90.00°
Resolution (Å)*	50-1.62 (1.65-1.62)*	50-1.76 (1.82-1.76)
*I/σ(I)*	58.9 (4.68)	17.2 (1.87)
*R_merge_*	0.069 (0.652)	0.111 (0.937)
Completeness (%)	99.94 (100.00)	95.89 (64.32)
Redundancy	12.7 (12.7)	8.7 (8.0)
**Refinement**		
Resolution (Å)	26.55 - 1.62 (1.678 - 1.62)*	36.95 - 1.762 (1.825 - 1.762)
No. reflections	13137 (1300)	20280 (1327)
*R*_work/_ *R*_free_	0.175/0.206	0.181/0.226
No. atoms		
Protein	826	1665
Ligand/ion	2	4
Water	75	270
*B*-factors		
Protein	30.10	25.87
Ligand/ion	67.00	40.03
Water	39.21	36.25
R.m.s. deviations		
Bond lengths (Å)	0.006	0.007
Bond angles (°)	0.83	0.87
Ramachandran plot(%)		
Favored	98.04	99.02
Allowed	1.96	0.98
Outliers	0.00	0.00

*Statistics for the highest-resolution shell are shown in parentheses.

The Ig-like domain of SARS-CoV-2 ORF8 (S84) forms a “β-sandwich” that comprises two β-sheets and an additional β-hairpin (**Figure 1B**). The smaller sheet is composed of three antiparallel strands (βB, βD, and βE), while the larger sheet is formed by five twisted strands (βA, βC, βC’, βF, βG) with loops (**Figures 1B, C**). The protruding β-hairpin exists between strands βC’ and βD, consisting of strands βI_1_ and βI_2_ (**Figure 1D**). It is the specific acquisition of a long insertion (residues 46 to 83) between strands C and D that distinguishes the ORF8 protein from other coronavirus Ig-like fold proteins, such as SARS-CoV-2 ORF7a (PDB ID: 7CI3) (12, 23). Of note, intramolecular disulfide bonds, including Cys25-Cys90, Cys37-Cys102, and Cys61-Cys83, contributed to stabilizing the β-sheet structure into an irregular β-sandwich fold.

Interestingly, unlike the solved SARS-CoV-2 ORF8 (L84) structure (PDB: 7JTL), the SARS-CoV-2 ORF8 (S84) contains only one molecule in an asymmetric unit. The electron density of residue Cys20 suggests that a potential intermolecular disulfide bond exists between two adjacent symmetric units (**Supplementary Figures 2A, B** **)**. Meanwhile, size-exclusion chromatography (SEC) analysis showed that ORF8 (S84) featured a dimeric form in the solution (**Supplementary Figure 2C**) (reducing and non-reducing SDS-PAGE, **Supplementary Figure 2D**). Accordingly, these results suggested that the SARS-CoV-2 ORF8 protein (S84) folded as a dimer (**Figure 1E**).

### Structural Overview and Alignment of RaTG13 ORF8

**Figure 2:**
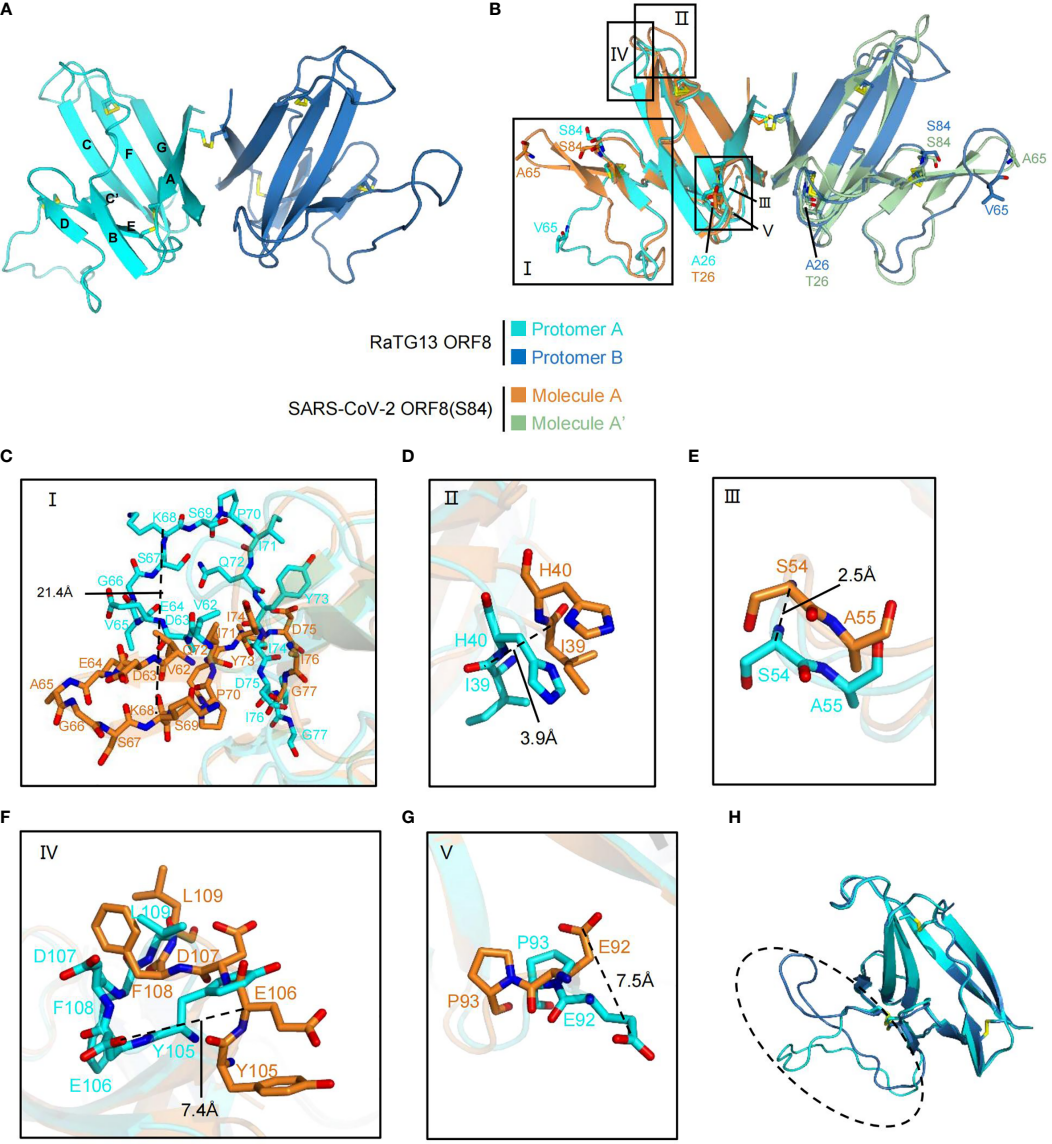
Comparison of RaTG13 ORF8 with SARS-CoV-2 ORF8 (S84). **(A)** Cartoon representation of the homodimer of RaTG13 ORF8 formed by Cys20 - Cys20 intermolecular disulfide bond. Protomer A is colored with cyan, and Protomer B is colored with blue. Disulfide bonds are presented in yellow with stick mode. The β-strands of protomer A are assigned with the alphabet from N-terminus to C-terminus. **(B)** Structural superimposition between RaTG13 ORF8 and SARS-CoV-2 ORF8 (S84). The diverse residues are shown with stick model and corresponded color. The structural differences of loops are shown in black boxes indicated with Roman numbers I to V. Box I, residues from 62 to 77; box II, residues from 39 to 40; box III, residues from 54 to 55; box IV, residues from 105 to 109; box V, residues from 92 to 93. **(C–G)** Details of structural differences from the superimposition of SARS-CoV-2 ORF8 (S84) molecule A and RaTG13 ORF8 protomer A. **(H)** Structural alignment between protomers A and B of RaTG13 ORF8. The residues 63 - 78 are highlighted in the dashed oval.

The Ig-like domain (16–121) of bat coronavirus RaTG13 ORF8 and SARS-CoV-2 ORF8 (S84) (this study) share 98% amino acid sequence identity (**Supplementary Figure 3**). To determine the sequence-structure relationship, we next solved the crystal structure of the RaTG13 ORF8 Ig-like domain at 1.76 Å, in which the electron density of residues 18-121 can be clearly traced (**Table 1**). It is worth noting that there are two molecules in one asymmetric unit, which is different from SARS-CoV-2 ORF8 (S84). Protomers A and B form a dimer *via* an intermolecular covalent Cys20-Cys20 disulfide bond. Similar to SARS-CoV-2 ORF8, the RaTG13 ORF8 Ig-like domain folds into a “β-sandwich” containing two β-sheets (strands βB, βD, and βE compose the small sheet; strands βA, βC, βC’, βF, and βG compose the large sheet) and an insertion region (**Figure 2A**). However, the insertion region forms a dynamic loop in the RaTG13 ORF8 Ig-like domain, distinguished from the β-hairpin fold in SARS-CoV-2 ORF8 (S84).

To obtain more detailed information on ORF8 variants, we superimpose the dimeric form of the SARS-CoV-2 ORF8 (S84) Ig-like domain structure with the RaTG13 ORF8 structure (**Figure 2B**). The root mean square deviation (RMSD) between these two structure coordinates is 0.861 Å over 208 superimposed Cα atoms (**Figure 2B**). Five dynamic regions were highlighted as Box I to V in superimposition between SARS- CoV-2 ORF8 molecule A and RaTG13 ORF8 protomer A (**Figure 2B** and **Supplementary Figure 4A**). Among them, the most different conformation change occurred in the residues 62-77 (Box I) of the insertion area between βC’-βD (**Figure 2C**). The residues from 62 to 77 in SARS-CoV-2 ORF8 (S84) assemble more tightly than their equivalent in RaTG13 ORF8, featuring a maximum 21.4 Å shift (**Figure 2C**). The electron density of residues 63-65 of RaTG13 ORF8 is unclear, indicating a dynamic region (**Supplementary Figure 4B**). The other differences are involved the conformational changes in the main chain, including the βB-βC loop 39 IH 40 (Box II) with 3.9 Å shift of residue Ile39 (**Figure 2D** and **Supplementary Figure 4C**), the βC-βC’ loop 54 SA 55 (Box III) with 2.5 Å shift of residue Ser54 (**Figure 2E** and **Supplementary Figure 4D**), and the βF-βG loop 105 YEDFL 109 (box IV) with 7.4 Å shift of residue Glu106 (**Figure 2F** and **Supplementary Figure 4E**). In addition, the βE-βF loop 92 EP 93 (box V) was shifting with 7.5 Å on the side chain of Glu92 (**Figure 2G** and **Supplementary Figure 4F**). Unlike SARS-CoV-2 ORF8 (S84), the residues 62-78 loop region of RaTG13 ORF8 is assembled different conformations between the protomer A and protomer B in one asymmetric unit (**Figure 2H**). In conclusion, the coronavirus ORF8 Ig-domain is formed by β-strands. The distinctive insertion region between strands βC and βD are dynamic, especially for the 62-78 residues.

### ORF8 Variants Form Homodimers *via* Cys20-Cys20 Intermolecular Disulfide Bond

**Figure 3:**
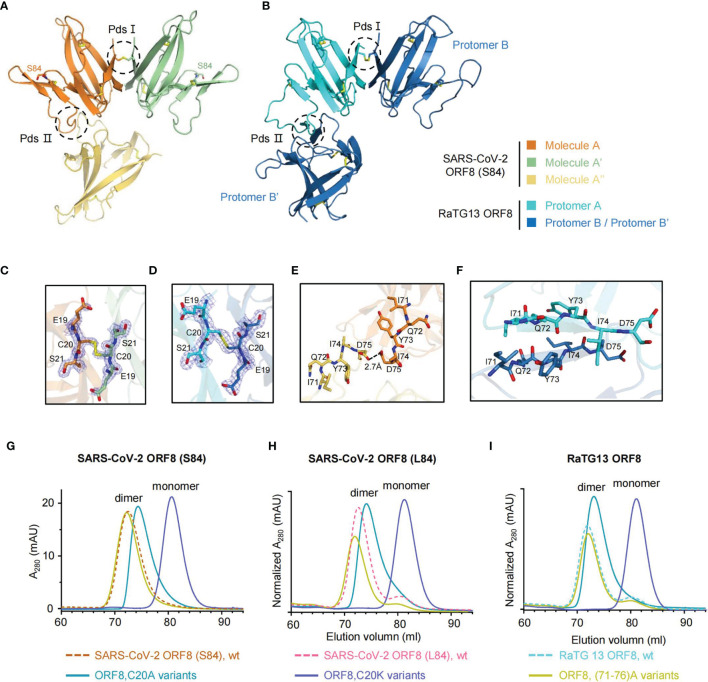
ORF8 forms a homodimer *via* Cys20-Cys20 intermolecular disulfide bond. **(A, B)** The symmetry mates of SARS-CoV-2 ORF8 (S84) **(A)** and RaTG13 ORF8 **(B)** are generated by PyMOL software, respectively. Putative dimer surfaces (Pds) are highlighted in dashed circles. PdsI, the C20-C20 covalent disulfide interface; PdsII, the putative noncovalent interface. **(C, D)** Stick representation of PdsI in SARS-CoV-2 ORF8 (S84) **(C)** and RaTG13 ORF8 **(D)** with 2Fo-Fc electron density map contoured at 1.2σ. Disulfide bonds are presented in yellow. **(E, F)** Stick representation of PdsII in SARS-CoV-2 ORF8 (S84) **(E)** and RaTG13 ORF8 **(F)**. **(G–I)** SEC analysis of SARS-CoV-2 ORF8 (S84) **(G)**, ORF8 (L84) **(H)**, and RaTG13 ORF8 **(I)** mutants (HiLoad 16/600 Superdex 75 pg column). The elution profiles of ORF8 mutant variants are shown as full-lines and and the ORF8 wild-type variants are shown as dash lines (HiLoad 16/600 Superdex 75 pg column). mAU, milliabsorption units. Normalized A280 indicated that the mAU values has normalized with input protein concentrations.

The dimeric forms of SARS-CoV-2 ORF8 variants, including the S84 and L84 isoforms, have been reported using prokaryotic or tobacco BY2 cell expression systems (11, 24, 25). The dimeric formation of ORF8 was found to be an evolutionary addition among SARS-CoV-2 and SARS2r-CoVs, distinguished from ORF7a and alpha-CoV Ig-like proteins (11, 12). A covalent disulfide bond is considered responsible for the ORF8 (L84) dimeric form, while it also may employ a noncovalent interface to contribute to other dimeric formation and higher-order assembly (11). However, the dynamic characteristics of the insertion region between strands βC’ and βD reveal that SARS-CoV-2 ORF8 (S84) form the dimer depending on the residue Cys20 rather than the other noncovalent interface. To identify this characteristic, we generated the symmetry mate of SARS-CoV-2 ORF8 (S84) (**Figure 3A**), ORF8 (L84) (**Supplementary Figure 5A**), and RaTG13 ORF8 (**Figure 3B**). The putative dimer surfaces (Pds) were highlighted with dash circles (**Figures 3A, B**). The Cys20-Cys20 interface was marked as Pds I, and the putative noncovalent interface was Pds II. In detail, the SARS-CoV-2 ORF8 (S84) dimer is linked by an intermolecular disulfide bond formed between the two Cys20 residues of molecule A and A’. (**Figure 3C**). Similar Cys20-Cys20 disulfide bond was found in SARS-CoV-2 ORF8 (L84) (PDB: 7JTL) (**Supplementary Figure 5B**) and RaTG13 ORF8 (this study) (**Figure 3D**). In contrast, residues 71-75 of protomer A form hydrophobic interactions with the corresponding residues of a symmetry-related copy of protomer B’ in PDB 7JTL in Pds II. The center of the interface forms a two-stranded parallel β-sheet (**Supplementary Figures 5A, C**). The 73YIDI76 motif was considered to stabilize the noncovalent dimer interface in the crystal (11). In ORF8(S84), however, the closest distance between the two 71 - 75 loops is 2.7 Å, which occurs at the side chain of the two Asp75 and may not help SARS-CoV-2 ORF8 (S84) to form a dimer with this interface (**Figure 3E**). Additionally, the RaTG13 ORF8 displayed a similar two-stranded parallel β-sheet in protomers A and B’, like SARS-CoV-2 ORF8 (L84) (**Figure 3F**).

To verify these observations, we performed mutagenesis analyses. The residue Cys20 was individually mutated to a charged lysine and small noncharged alanine in ORF8 variants, including SARS-CoV-2 ORF8 (S84), ORF8 (L84), and RaTG13 ORF8. In size-exclusion chromatography (SEC), all the C20K mutations of ORF8 variants became monomers (**Figures 3G–I**). Based on the crystal structure (PDB 7JTL), the complementary electrostatic potential of the interfaces and the intermolecular bonds closed surrounding Cys20 were also supposed to stabilize the covalent dimer (11). Of note, the small and noncharged mutation C20A have lower molecular weight shifts in the elution peak of the SEC chromatogram (**Figures 3G–I**). The transformation of charged lysine mutation indicated that Cys20 is the critical residue to form a dimer.

To go further clarification of the inter-chain disulfide bond by Cys20, we apply the reducing [supplemented with DL-dithiothreitol (DTT)] and non-reducing SDS-PAGE on the SARS-CoV-2 ORF8(S84), SARS-CoV-2 ORF8(S84) C20A mutant, SARS-CoV-2 ORF8(S84) C20K mutant (**Supplementary Figure 5D**). The SARS-CoV-2 ORF8(S84) form a stable dimer in the absence of the reducing reagent. By contrast, C20A and C20K mutants could not form a stable dimer in the absence of the reducing reagent. Thus, these data suggest that the SARS-CoV-2 ORF8(S84) form an inter-chain disulfide bond at Cys20. Similarly, the mutants of SARS-CoV-2 ORF8 (L84) and RaTG13 ORF8 display similar characteristics under non-reducing conditions (**Supplementary Figures 5E, F**).

We also mutated 71 IQYIDI 76 to 71 AAAAAA 76 in ORF8 variants [termed as (71–76)A]. The SEC analysis showed that the SARS-CoV-2 ORF8 (S84) (71–76) A mutant maintained the dimeric form (**Figure 3G**). The results were similar in the context of SARS-CoV-2 ORF8 (L84) (71–76) A and RaTG13 ORF8 (71–76) A protein (**Figures 3H, I** **)**. Hence, our data suggested that residues 71 IQYIDI 76 may not contribute to the dimerization of ORF8 under non-reducing condition.

### SARS-CoV-2 ORF8 Interacts With Human CD14^+^ Monocytes

**Figure 4:**
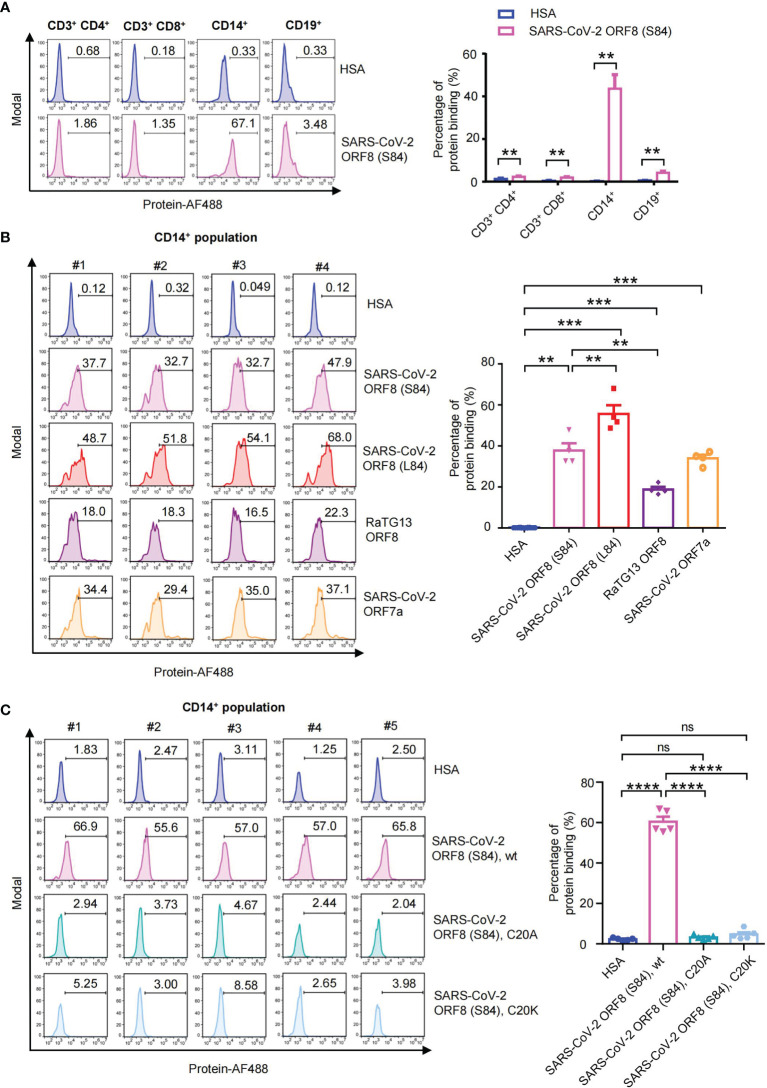
Human PBMC binding assay of SARS-CoV-2 Ig-like domain protein. **(A)** Alexa Fluor 488-coupled SARS-CoV-2 ORF8 (S84) incubated with human PBMCs from healthy donors (n = 5), and the binding abilities to CD3^+^ CD4^+^ T cells, CD3^+^ CD8^+^ T cells, CD14^+^ monocytes and CD19^+^ B cells were analyzed *via* flow cytometry. Representative flow cytometry plots (left) and statistical analysis (n=5) (right) are present. **(B)** Alexa Fluor 488-coupled proteins incubated with human PBMCs from healthy donors (n = 4), and their binding abilities to CD14^+^ monocytes were analyzed *via* flow cytometry. Human serum albumin (HSA), SARS-CoV-2 ORF8 (L84), SARS-CoV-2 ORF8 (S84), Bat coronavirus RaTG13 ORF8, SARS-CoV-2 ORF7a are present. **(C)** Alexa Fluor 488-coupled proteins incubated with human PBMCs from healthy donors (n = 5), and their binding abilities to CD14^+^ monocytes were analyzed *via* flow cytometry. Human serum albumin (HSA), SARS-CoV-2 ORF8 (S84), and its C20A, C20K mutant protein are present. Data were analyzed using paired two-tailed Student’s t-tests, and the error bars show the means ± SEM ( **P < 0.01; ***P < 0.001; ****P < 0.0001; ns, nonsignificant).

SARS-CoV-2 ORF8 is an Ig-like domain protein and has immune-related functions in humans. SARS-CoV-2 ORF8 was characterized as a secreted protein detected in the cell culture supernatant and sera of COVID-19 patients (14). To figure out whether coronavirus ORF8 has the binding capability to immune cells, we perform the immune cell-binding assays. With peripheral blood mononuclear cell samples, we validated the abilities of SARS-CoV-2 ORF8 (S84) interacting with human CD3^+^ CD4^+^ T cells, CD3^+^ CD8^+^ T cells, CD14^+^ monocytes, and CD19^+^ B cells, respectively. We used human serum albumin (HSA) as a negative control, which cannot interact with these immune cells (23). Luckily, we found that SARS-CoV-2 ORF8 (S84) interacts with CD14^+^ monocytes with a significant difference compared to HSA, while weak signals were detected on CD3^+^ CD4^+^ T cells, CD3^+^ CD8^+^ T cells, CD19^+^ B cells (**Figure 4A**). Next, we assessed the abilities of SARS-CoV-2 ORF8 (S84), SARS-CoV-2 ORF8 (L84), and RaTG13 ORF8 to interact with human CD14^+^ monocytes. We had confirmed that SARS-CoV-2 ORF7a has the binding capability to human CD14^+^ monocytes in our previous work. Therefore, the SARS-CoV-2 ORF7a was used as a positive control in these assays (23). Intriguingly, ORF8 variants have different interaction efficiencies. SARS-CoV-2 ORF8 (L84) was the strongest, followed by SARS-CoV-2 ORF8 (S84), and RaTG13 ORF8 was the last (**Figure 4B**). These results suggest that ORF8 predominantly interacts with human CD14^+^ monocytes, and its variants display diverse potency in binding to immune cells. To further explore the characteristics of dimeric form ORF8, we analyzed the binding abilities of SARS-CoV-2 ORF8 (S84), C20A, C20K protein with these assays. As shown in **Figure 4C**, the dimeric SARS-CoV-2 ORF8 (S84) was the strongest binding partner to CD14^+^ monocytes, compared with the SARS-CoV-2 ORF8 (S84) mutants.

## Discussion

ORF8 was identified as one of the fast-evolving genomic regions in SARS-CoV-2 and related viruses. Herein, we report the 1.62 Å crystal structures of SARS-CoV-2 ORF8 (S84) and 1.76 Å crystal structures of bat coronavirus RaTG13 ORF8. The superimposition of SARS-CoV-2 ORF8 (S84) with RaTG13 ORF8 indicates a very similar β-stands formation. However, the insertion region is highly dynamic compared with equivalent residues between SARS-CoV-2 ORF8 (S84) and RaTG13 ORF8, especially 62 to 77 amino acids, which can adopt different conformations. In the crystal structures of SARS-CoV-2 ORF8 (S84) and RaTG13 ORF8, the conformation diversities of insertion region between stands βC’-βD may be due to crystal packing. However, the non-reducing SDS-PAGE results of mutants containing 71 AAAAAA 76 indicated that residues 71-76 are dispensable for dimerization under solvent conditions (**Supplementary Figures 5D–F**). From this aspect, the insertion region may not contribute to the overall structure stabilization but suggests the potential interaction to adapt different ligands. In addition, the variant positions (including residues 26, 65 and 84) may influence the neighborhood residues’ electron density and ultimately result in structural-functional differences. Residues 65 may influence the electron density surrounding it. For example, the electron density of Asp63 and Glu64 in RaTG13 protomer A are unclear (**Supplementary Figure 4B**). The electron density of Ala65 and Gly66 of SARS-CoV-2 ORF8 (L84) PDB:7JTL are missing (**Supplementary Figure 6A**). We applied the superimposition of diverse residues 26 on SARS-CoV-2 ORF8 (S84), SARS-CoV-2 ORF8 (L84), and RaTG13 ORF8 (**Supplementary Figure 6B** **)**, and observed residues 26 are next to the Cys25-Cys90 disulfide bond. Similarly, residues 84 are next to the disulfide-forming Cys83. Meanwhile, the Cys61-Cys83 disulfide bond is flanked by the insertion area, which adopts the most different conformation change that occurred in residues 62–77 (Box I). Furthermore, an unusual *cis* conformation Pro85 was observed on the other side of residues 84 **(** **Supplementary Figure 6C** **)**. Even though residues 26 and 84 are both exposed to solvent, the unusual variant positions (including residues 26, 65, and 84) may influence the neighborhood disulfide bonds and ultimately result in functional differences. In accordance with our result, the insertion region between stands βC’-βD has been shown as a dynamic region in another unpublished SARS-CoV-2 ORF8 (L84) structure (PDB: 7JX6). Recently study has reported that ORF8 (L84) and ORF8 (S84), using the tobacco BY-2 cell production system, showed different biochemical characteristics, as evaluated by NMR (25).

Complex immune responses are closely involved in the development of severe COVID-19, and disordered inflammation may be responsive to the sudden deterioration of COVID-19 patients (26). This inflammatory response is characterized by elevated blood concentration of pro-inflammatory cytokines, such as interleukin-6 (IL-6), granulocyte-macrophage colony-stimulating factor (GM-CSF), and tumor necrosis factor alpha (TNFα) (27, 28). Monocyte sensing of SARS-CoV-2 and the subsequent secretion of pro-inflammatory cytokines have been reported to be critical for this dysregulated inflammatory response (29). However, the exact mechanisms that mediate the initial interaction of the monocytes and SARS-CoV-2 remain ill-defined.

To further investigate the consequences of the viral proteins, potentially diverse protein functions were studied. We detected the abilities of ORF8 variants to interact with the multiple immune cells from healthy human PBMCs. Strong interactions were found between the ORF8 proteins with CD14^+^ monocytes. In contrast, weaker signals were detected on CD3^+^ CD4^+^ T cells (**Supplementary Figure 7**), CD3^+^ CD8^+^ T cells (**Supplementary Figure 8**), and CD19^+^ B cells (**Supplementary Figure 9**). In particular, SARS-CoV-2 ORF8 (S84), ORF8 (L84), and RaTG13 ORF8 differ in their direct interactions with CD14^+^ monocytes. Significantly, the hyperinflammatory subtype of monocytes express CD14 and CCL3, which broadly express more cell-type-specific cytokines, and this might be one of the major sources in PBMCs triggering the inflammatory cytokine storm in severe COVID-19 patients (19). Our previous study identified that SARS-CoV-2 ORF7a interacts with CD14^+^ monocytes, leading to a significant decrease in the antigen-presenting-related cell surface molecules HLA-DR/DP/DQ on CD14^+^ monocytes. SARS-CoV-2 ORF7a coincubation with monocytes triggered upregulation of the pro-inflammatory cytokines, including IL-6, IL-1β, IL-8, and TNF-α, which are the abundant cytokines detected in SARS-CoV-2 infection (28, 30, 31). We presume that ORF8 cooperates with ORF7a to affect the antigen-presenting ability of these monocytes and then regulates the immune response. ORF8 may be responsible for monocyte sensing of SARS-CoV-2 and the subsequent cytokine storm of COVID-19. The mild disease outcome related S84 variant of the ORF8 protein demonstrates weaker binding ability with CD14^+^ monocytes (**Figure 4**), indicating that the ORF8 variants may adjust different degrees of inflammation by modulating monocyte recognition of viral antigens. Moreover, we identified the Cys20 is the critical residue for the ORF8 dimeric form and immune cell binding. Residue Cys20 and its immediately surrounding residues are conserved in the most recent bat orthologues (RaTG13 and SL-CoVZC45) of SARS-CoV-2 ORF8. However, Cys20 is not conserved in ORF8 of SARS-CoV, and most of its related bat beta-coronavirus such as YNLF_31C and BtRf BetaCoV-HeB2013. Hence, the ORF8 covalent dimer was considered as an evolutionarily recent addition among human beta-coronaviruses unique to SARS-CoV-2 (11). SARS-CoV-2 ORF8 dimer-related positions may be potential antiviral agent-specific targets.

In general, the structures suggest that SARS-CoV-2 ORF8 (S84) and the closely related RaTG13 ORF8 employed patterns for the dimer and indicate that the function of the residue 26, 65, 84 variant sites to stabilize the tertiary structure may relate to the interaction with human CD14^+^ monocytes. Our data provide a new strategy for developing coronavirus access protein-based immune interventions.

## Data Availability Statement

The structures for this study can be found in the Protein Data Bank with 7F5F and 7F8L access codes.

## Author Contributions

SC, JuC, and JL contributed to the conception of the study and established the construction of the article. SC, JuC, XC, CH, ZCZ, and ZLZ designed the experiments. XC, ZCZ, CH, SC, JL, and JuC drew the figures and wrote the manuscript. XC and ZCZ contributed to plasmid cloning, protein purification, and crystallization. CH carried out the cellular experiments. SC, XC, and ZCZ performed the structural determination and validation. ZXH prepared the materials for the experiments. CH, GJ, and ZSH contributed to clinical samples collections. ZLZ, SK, QC, MY, SH, SL, JiC, KL, and XL helped to analyze the experimental data. XC, ZCZ, and CH contributed equally to this work. All authors contributed to the article and approved the submitted version.

## Funding

This work was supported by project grants from the National Key R&D Program of China (2019YFA0110300 and 2020YFA0509400) to JC; the National Natural Science Foundation of China (No. 32171192 and No. 31770801) to SC; the Natural Science Foundation of Guangdong Province, China (No. 2018B030306029) to SC; the National Natural Science Foundation of China (82071745) to JC; the Science and Technology Program of Guangzhou to JC (202002030069); the Guangdong Project (2019QN01Y212) to JC.

## Conflict of Interest

The authors declare that the research was conducted in the absence of any commercial or financial relationships that could be construed as a potential conflict of interest.

## Publisher’s Note

All claims expressed in this article are solely those of the authors and do not necessarily represent those of their affiliated organizations, or those of the publisher, the editors and the reviewers. Any product that may be evaluated in this article, or claim that may be made by its manufacturer, is not guaranteed or endorsed by the publisher.
