# Supplementary material for: Crystal Structures of Bat and Human Coronavirus ORF8 Protein Ig-Like Domain Provide Insights Into the Diversity of Immune Responses
Source: Front Immunol. 2021 Dec 17;12:807134. doi: 10.3389/fimmu.2021.807134 (PMC8718552; doi:10.3389/fimmu.2021.807134)
Supplement: Supplementary file 1 [file DataSheet_1.pdf]

Supplementary Material

1. Supplementary Figures

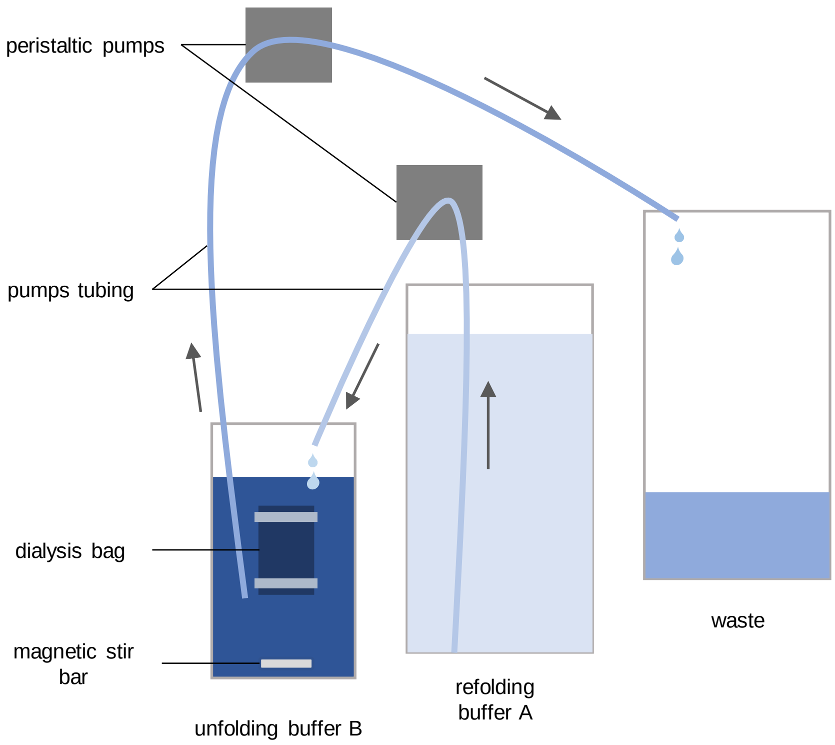

Supplementary Figure 1. Schematic drawing of the experimental method of refolding with salt gradient dialysis.

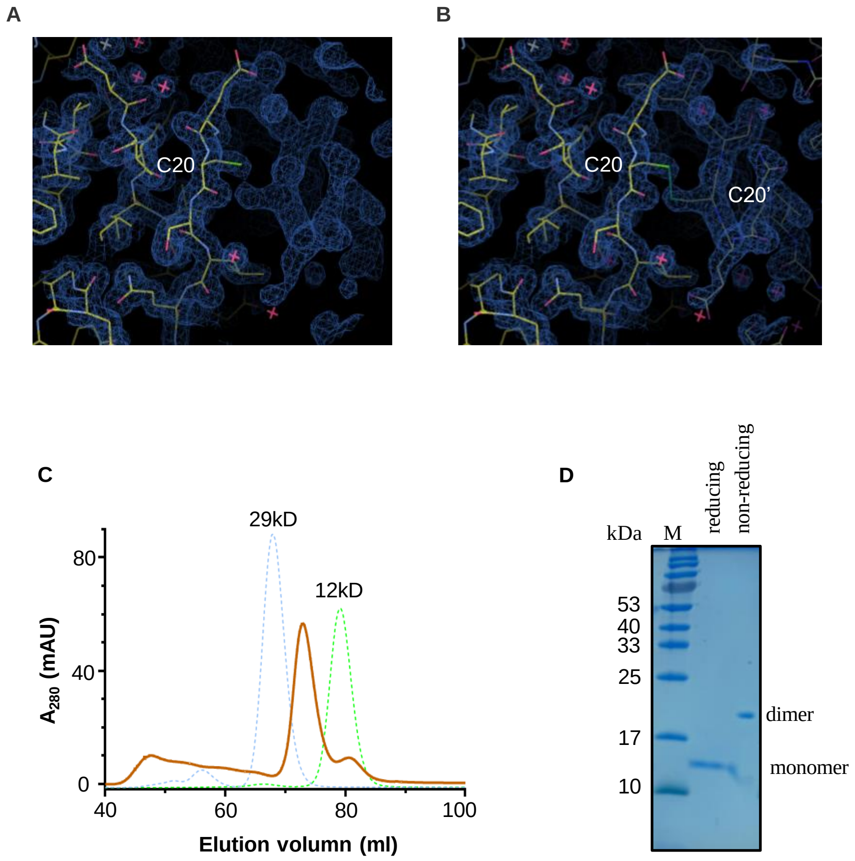

Supplementary Figure 2 . (A) A zoomed view of the 2Fo-Fc electron density of SARS-CoV-2 ORF8 (S84). The electron density map contoured at 1.2σ. The residues Cys20 of protomer A is shown. **(B)** The symmetry mates of **(A)** were showed, and the Cys20-Cys20’ disulfide bond was present. **(C)** SEC analysis of SARS-CoV-2 ORF8 (S84) dimer (orange full line), Carbonic Anhydrase (blue dashed line) and Cytochrome c (green dashed line) as markers (HiLoad 16/600 Superdex 75 pg column). **(D)** The reducing and non-reducing SDS-PAGE of Ig-domains of SARS-CoV-2 ORF8 (S84).

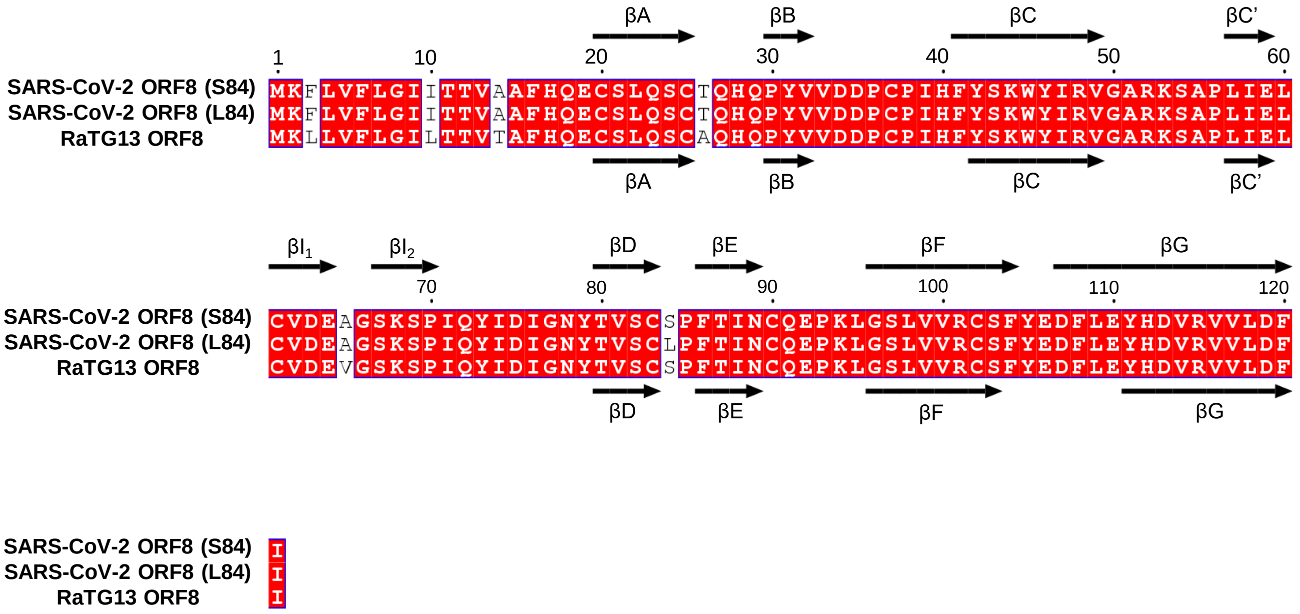

Supplementary Figure 3. Multiple sequence alignment of SARS-CoV-2 ORF8 (S84), SARS-CoV-2 ORF8 (L84) and RaTG13 ORF8. Secondary structure assignments (black arrows) correspond to ORF8 structures. Up, SARS-CoV-2 ORF8 (S84); bottom, RaTG13 ORF8.

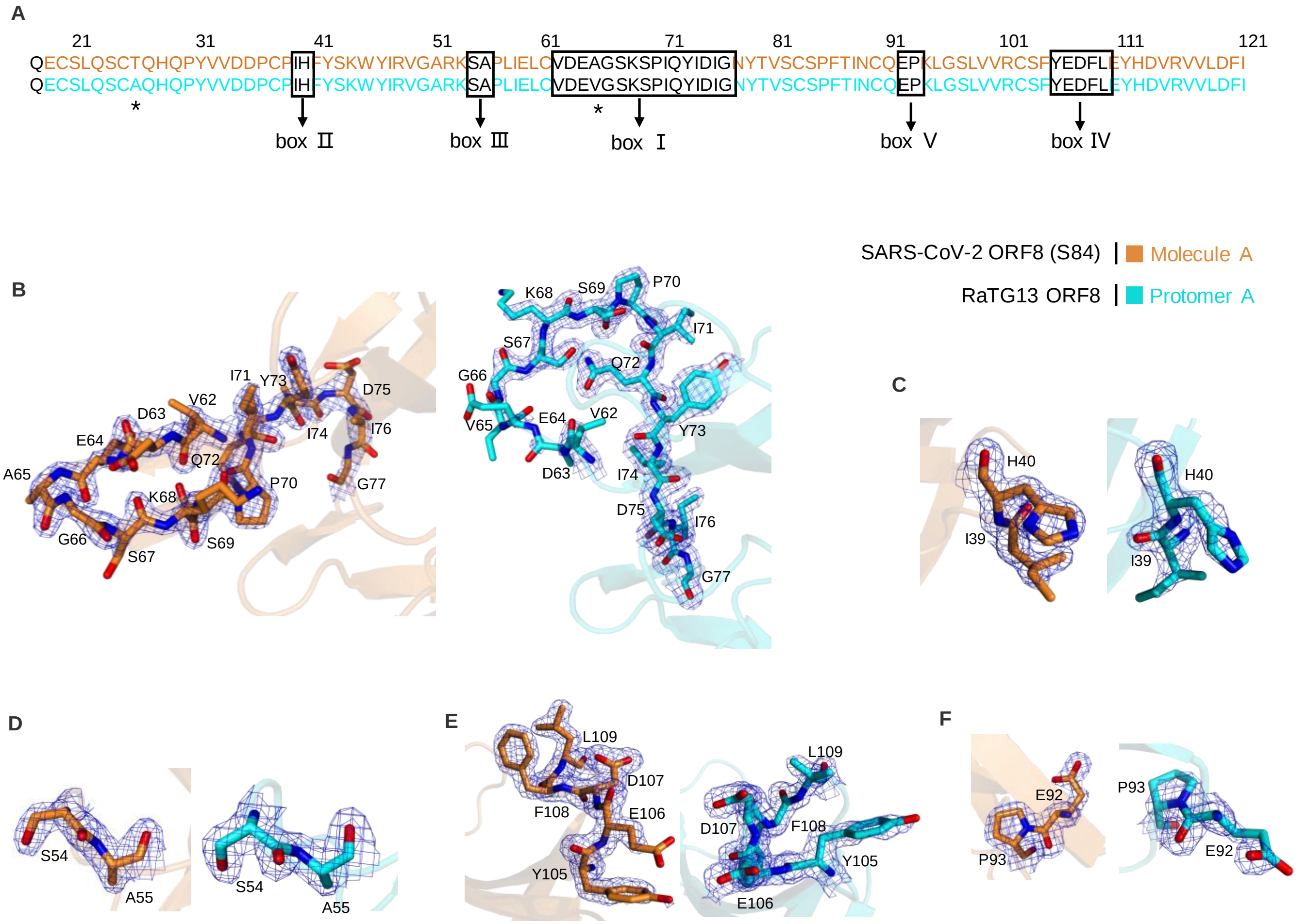

Supplementary Figure 4. Structural difference sites between SARS-CoV-2 ORF8 (S84) molecule A and RaTG13 ORF8 protomer A. **(A)** Sequence alignment of the SARS-CoV-2 ORF8 (S84) molecule A and RaTG13 ORF8 protomer A. The structural difference sequences are highlighted in black and solid line box I to V. The diverse residues are marked with asterisks. **(B)** – **(F)** The corresponding 2Fo-Fc electron density of superimpose structural difference sites (box I to V shown in **Figure 2**) between SARS-CoV-2 ORF8 (S84) molecule A and RaTG13 ORF8 protomer A. The electron density maps are all shown at a contour level of 1.2σ.

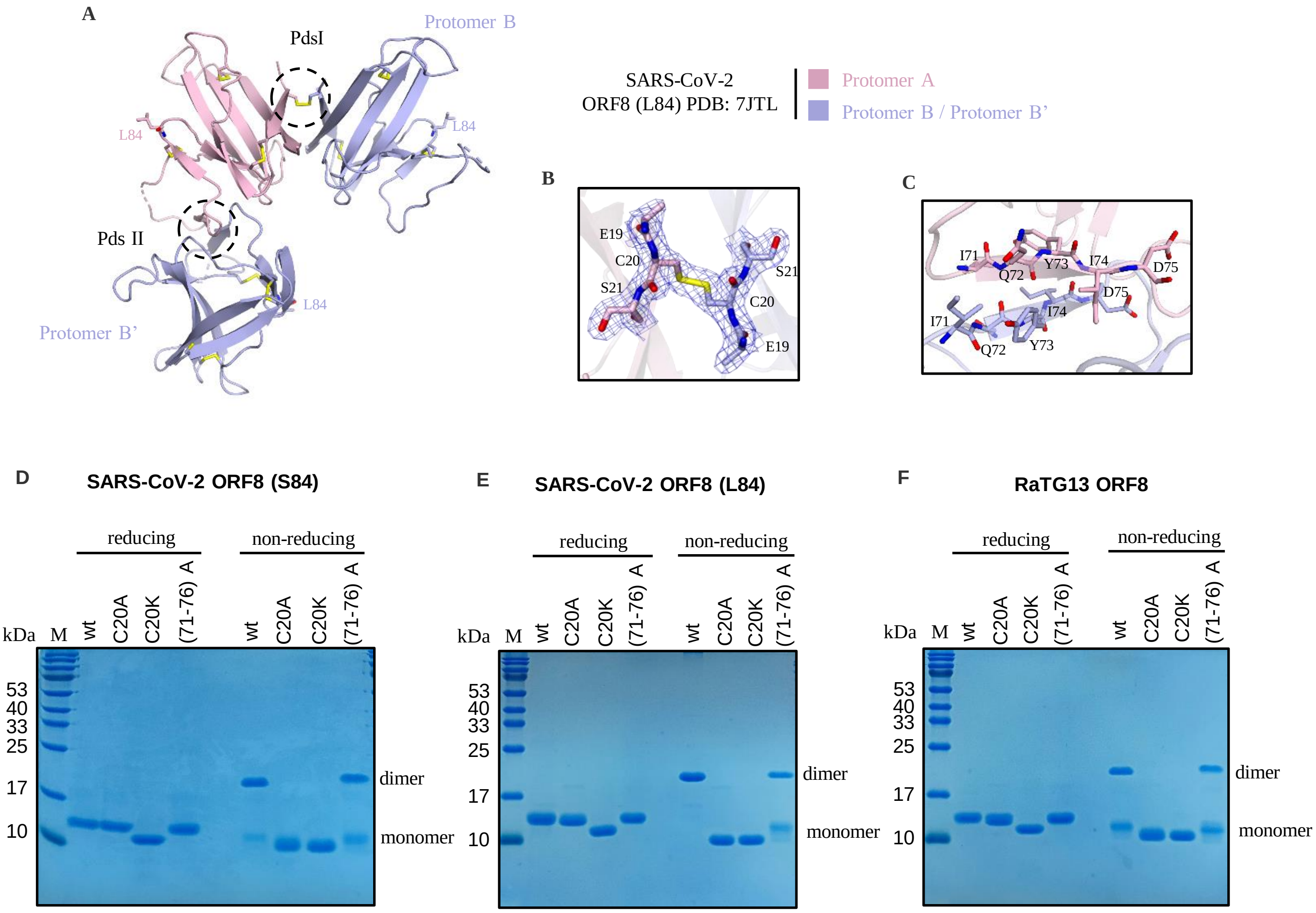

Supplementary Figure 5. ORF8 protein forms a homodimer via Cys20-Cys20 intermolecular disulfide bond. **(A)** The symmetry mate of SARS-CoV-2 ORF8 (L84) (PDB: 7JTL) was generated by PyMOL software. Putative dimer surface (Pds) are highlighted in dashed cirlces. Pds I, the Cys20-Cys20 covalent disulfide interface; Pds II, the putative noncovalent interface. **(B)** Stick representation of PdsI in SARS-CoV-2 ORF8 (L84) with 2Fo-Fc electron density map contoured at 1.2σ. Disulfide bonds are presented as yellow sticks. The side chains of PDB 7JTL Glu19 do not show because of the density lacking. **(C)** Stick representation of PdsII in SARS-CoV-2 ORF8 (L84). (**D**) – (**F**) The reducing and non-reducing SDS-PAGE of Ig-domains of SARS-CoV-2 ORF8 (S84) (**D**), SARS-CoV-2 ORF8 (L84) (**E**), and RaTG13 ORF8 (**F**) mutants. wt, wild-type; M, molecular weight marker. Nombers represent molecular weights (kDa).

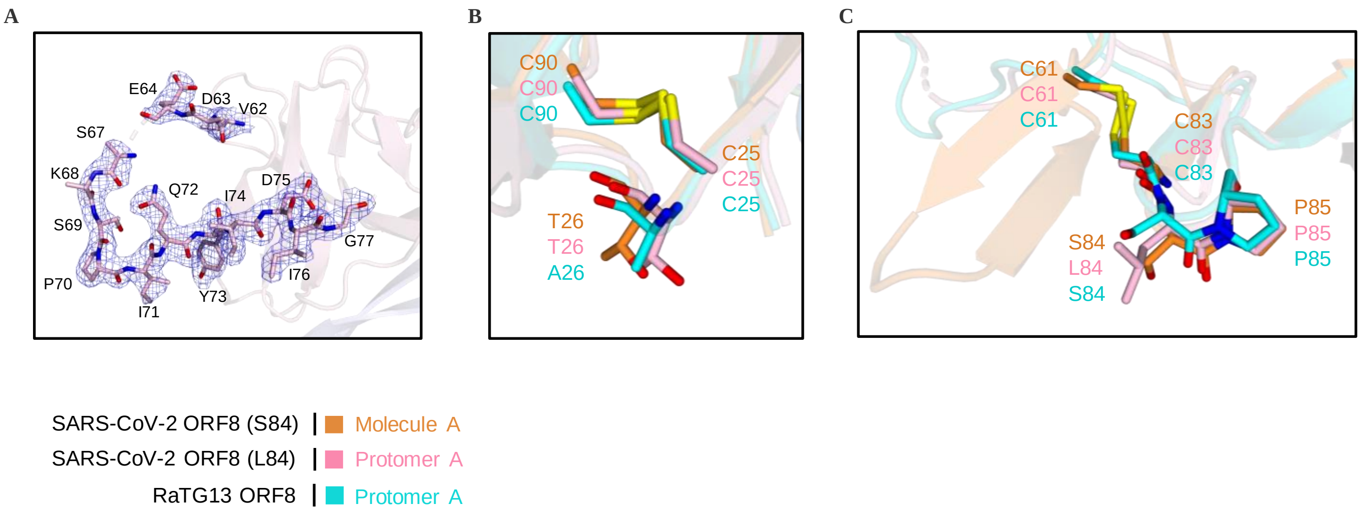

Supplementary Figure 6 . (A) The corresponding 2Fo-Fc electron density of SARS-CoV-2 ORF8 (L84) PDB:7JTL. The electron density maps are all shown at a contour level of 1.2σ. **(B) (C)** Structural superimposition among SARS-CoV-2 ORF8 (S84), SARS-CoV-2 ORF8 (L84) and RaTG13 ORF8. The diverse residues and disulfide bond are shown as sticks.

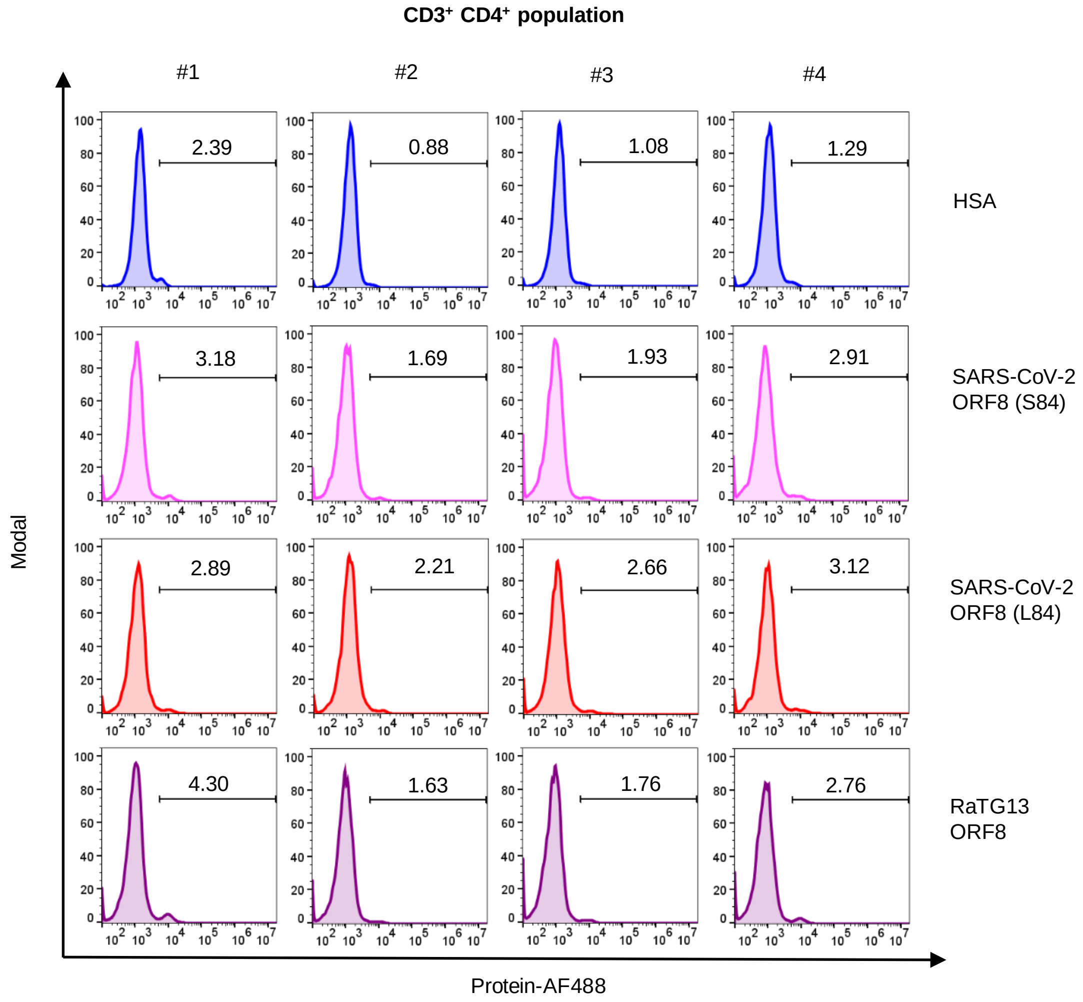

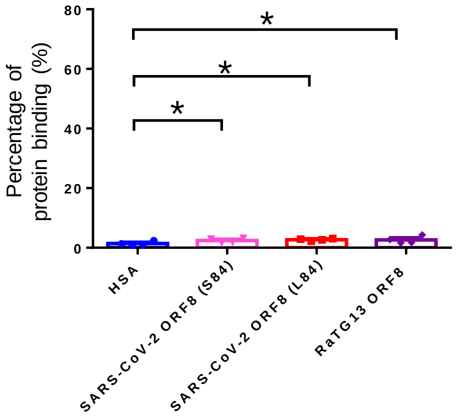

Supplementary Figure 7. Human PBMC source CD3$^+$ CD4$^+$ T cells binding assay of SARS-CoV-2 and RaTG13 Ig-like domain proteins (n=4). Data were analyzed using paired two-tailed Student's t-tests, and the error bars show the means ± SEM (*, $P < 0.05$).

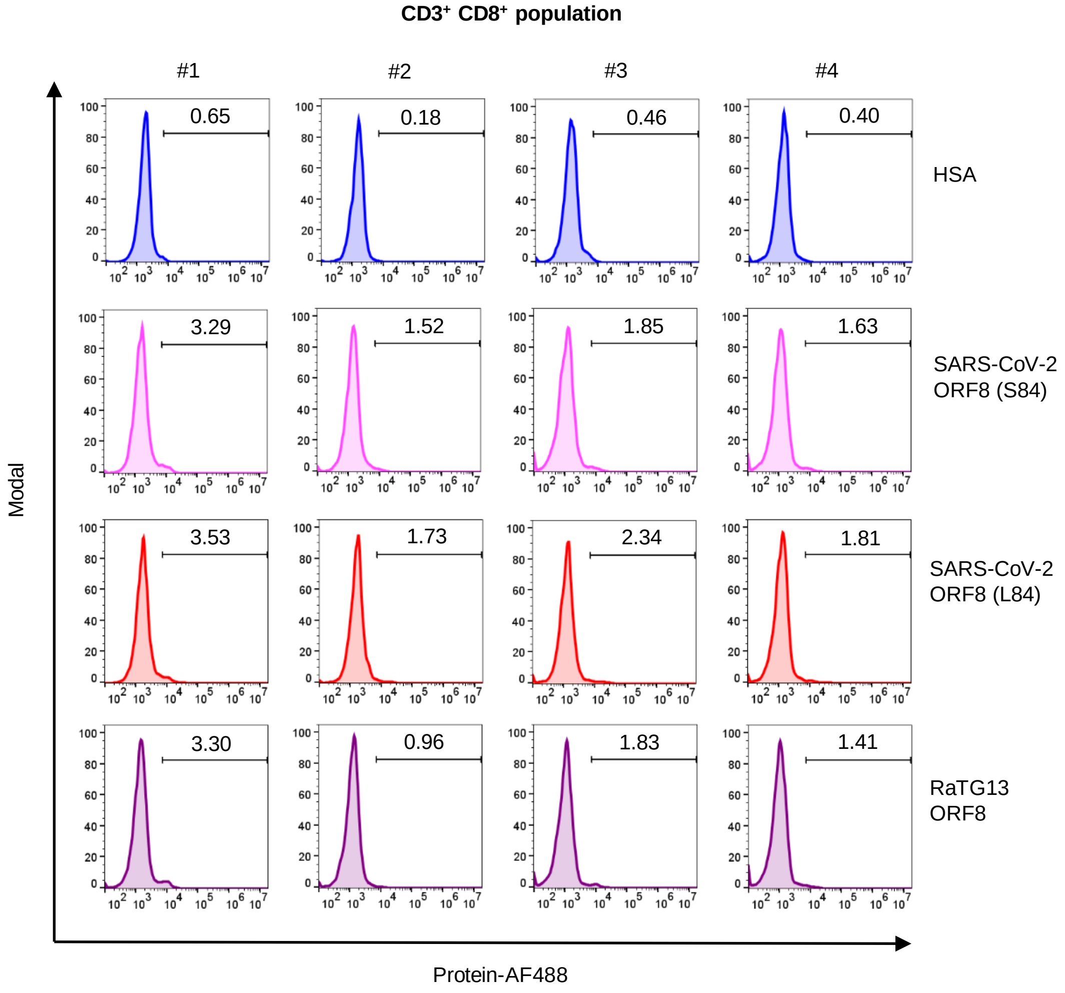

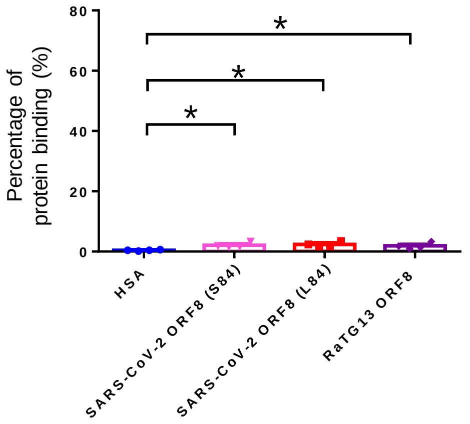

Supplementary Figure 8. Human PBMC source CD3$^+$ CD8$^+$ T cells binding assay of SARS-CoV-2 and RaTG13 Ig-like domain proteins (n=4). Data were analyzed using paired two-tailed Student's t-tests, and the error bars show the means ± SEM (*, $P < 0.05$).

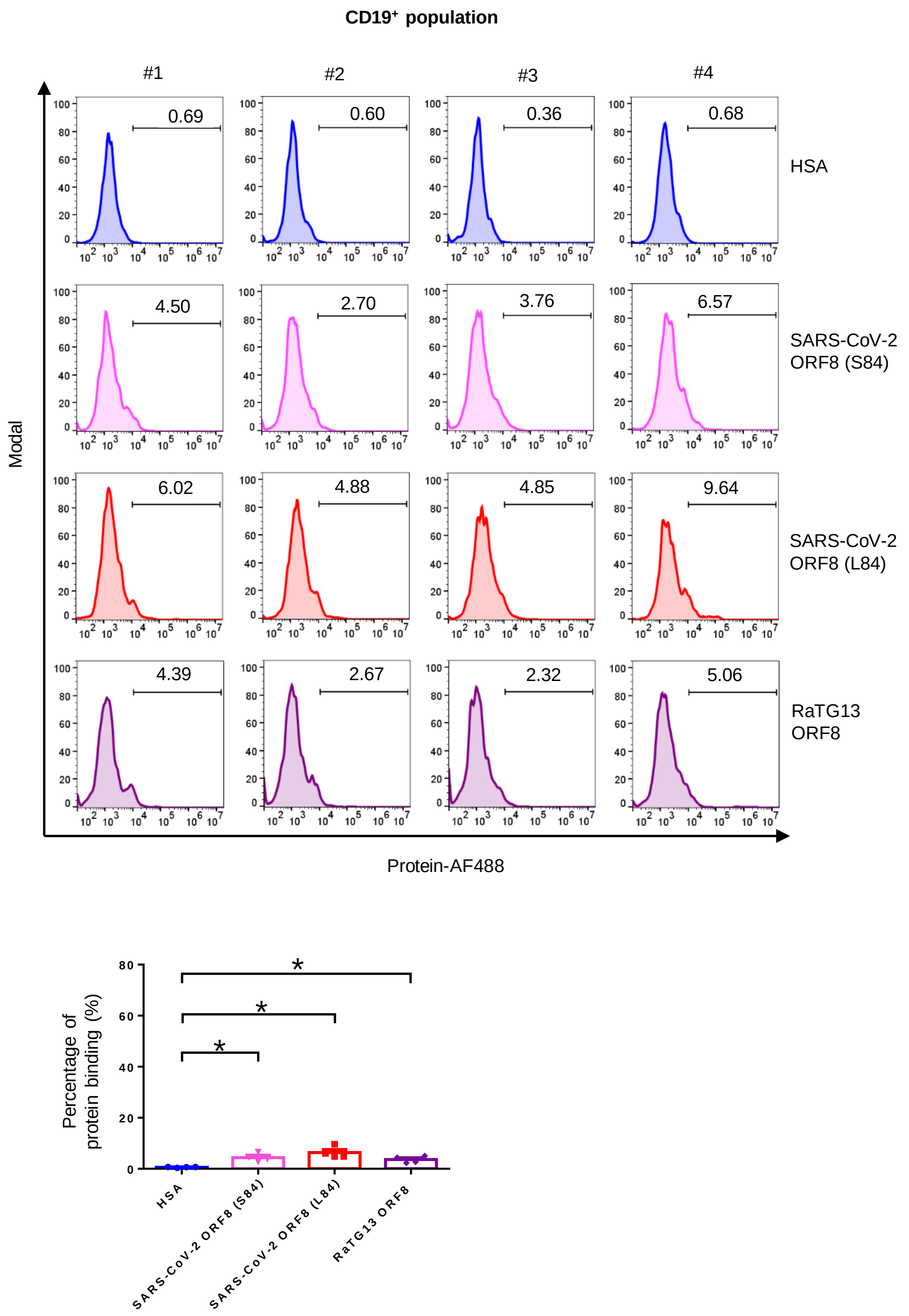

Supplementary Figure 9. Human PBMC source CD19+ B cells binding assay of SARS-CoV-2 and RaTG13 Ig-like domain proteins (n=4). Data were analyzed using paired two-tailed Student's t-tests, and the error bars show the means ± SEM (*, $P < 0.05$).
